# Meckel’s Diverticulum Causing a Small Bowel Obstruction in an 80-Year-Old Male: Report of a Rare Case and Review of the Literature

**DOI:** 10.7759/cureus.35990

**Published:** 2023-03-10

**Authors:** Johnny Zaatar, Souad Ghattas, Jad Al Bitar, Raja Wakim

**Affiliations:** 1 Medicine and Medical Sciences, University of Balamand, Beirut, LBN; 2 General Surgery, University of Balamand, Mount Lebanon Hospital University Medical Center, Beirut, LBN

**Keywords:** meckel´s diverticulum, small bowel resection, elderly patient, virgin abdomen, small-bowel obstruction, meckel's diverticulum in adults

## Abstract

Meckel’s diverticulum (MD) is the most common congenital malformation occurring in the gastrointestinal tract and results from the persistence of the vitelline duct during embryology. MD is typically asymptomatic in adults with most of its symptoms manifesting in early childhood. Small bowel obstruction (SBO) due to MD in the elderly population is an entity that has not been widely described in the literature. We present a very rare case of SBO in an 80-year-old patient with no previous abdominal surgeries (virgin abdomen). The cause of obstruction was determined to be an adhesive band formed on top of an MD. The obstruction was relieved and the small bowel segment that contained the diverticulum was resected, and anastomosis was made.

## Introduction

Small bowel obstruction (SBO) is a common cause of acute abdominal pain associated with nausea, vomiting and abdominal distention. SBO requires urgent hospitalization and evaluation to prevent serious complications [1]. Adhesions are the most common cause of SBO and are generally acquired after pelvic or gastrointestinal surgeries [1]. However, adhesions can also arise from congenital or inflammatory conditions, such as Meckel’s diverticulum (MD) [1,2]. MD is the most common congenital malformation occurring in the gastrointestinal tract and results from the persistence of the vitelline duct during embryology [3]. MD is typically asymptomatic in adults with most of its symptoms manifesting in early childhood. SBO can be a rare complication of MD frequently due to fibrous bands of adhesion forming around the diverticulum [2-5]. We present a rare case of SBO occurring in an elderly patient with no previous history of gastrointestinal surgery, and in whom, surgical exploration showed that an adhesion band has formed on top of an MD and caused the SBO.

## Case presentation

This is the case of an 80-year-old male with a previous medical history of hypertension, diabetes mellitus type II, and dyslipidemia well controlled respectively with antihypertensive, antidiabetic medications, and statins. The patient presented to the emergency department at our institution with abdominal pain for four days duration. The pain was crampy, colicky, acute in onset, and generalized but non-radiating. The pain was associated with constipation, obstipation, and recurrent vomiting which led to decreased oral food intake. The patient denied hematochezia, hematemesis, melena, diarrhea, weight loss, or decreased appetite. The patient also denied any exacerbating or relieving factors and denied any previous abdominal surgery. Significant findings on the physical exam included a distended abdomen, hypoactive bowel sounds, tympany upon percussion, and minimal tenderness to palpation without guarding or rebound tenderness. Laboratory findings on admission include white blood cell count = 7.39 x 10^9^/L (neutrophils 72%, lymphocytes 9.3%), hemoglobin = 11.4 g/dL, hematocrit = 33.3%, platelets = 233,000 per microliter, sodium = 136 mmol/L, potassium = 5.6 mmol/L, chloride = 99 mmol/L, carbon dioxide = 21 mmol/L, creatinine = 2.85 mg/dL, and C-reactive protein = 11 mg/L.

**Figure 1:**
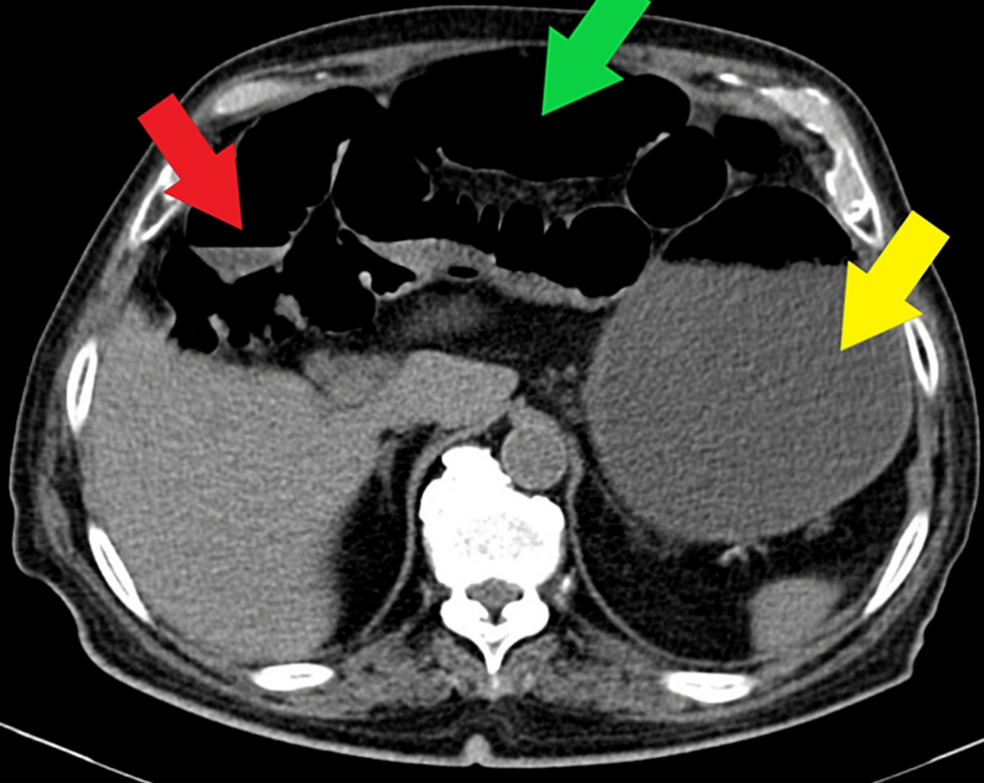
Non-contrast CT scan of the abdomen showing air fluid levels, distended small bowels, and an enlarged stomach suggesting an obstruction. Red arrow: air-fluid levels; Green arrow: distended small bowels; Yellow arrow: enlarged stomach CT: Computed Tomography

**Figure 2:**
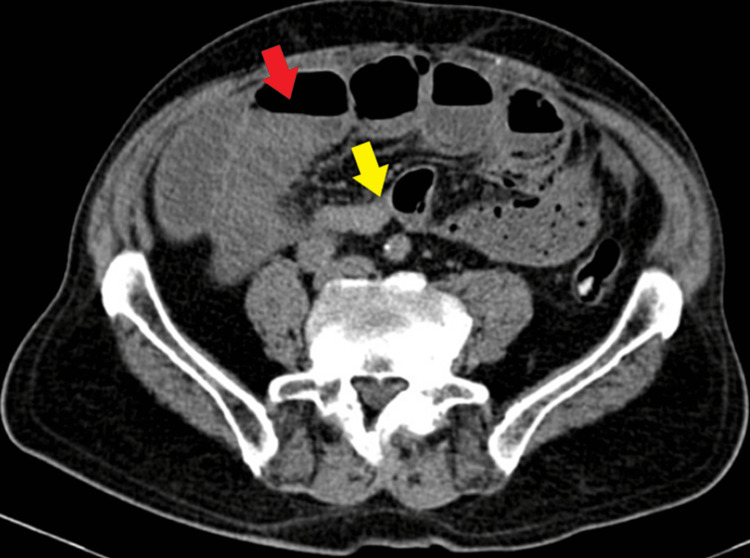
Non-contrast CT scan of the abdomen showing air fluid levels in the small bowels and a transitional zone in the distal ileum at the midline infraumbilical location. Red arrow: air-fluid levels; Yellow arrow: transition zone CT: Computed Tomography

On admission, a computed tomography (CT) scan of the abdomen and pelvis without intravenous contrast showed dilated small bowels with air-fluid levels and a transitional zone in the distal ileum at the midline infraumbilical location. The colon contained fecal residue with air and the stomach was large and distended. There were no signs of bowel compromise. The findings suggested a diagnosis of bowel SBO (Figures 1, 2).

**Figure 3:**
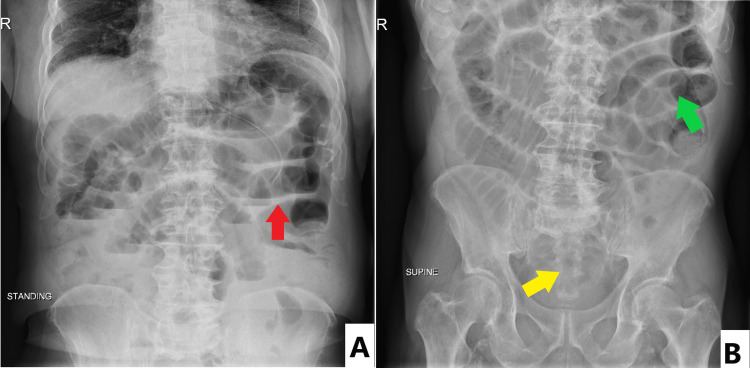
Abdominal x-ray in the standing (A) and supine (B) positions showing air-fluid levels in the standing position and distended small bowel loops in the supine position. The absence of air in the large bowels and rectum also suggest an obstruction in the small bowels. Red arrow: air-fluid levels; Green arrow: distended small bowels; Yellow arrow: absence of air in rectum

Nasogastric tube decompression was performed with an output of 500 mL of bile-stained liquid secretions. Based on the clinical and imaging findings, the diagnosis was SBO probably due to adhesions. Since there were no indications for immediate surgery (the CT scan showed no signs of bowel ischemia, necrosis, perforation, closed-loop obstruction, or localized small bowel tumor), the decision was made for initial conservative management with a plan for surgery if the obstruction does not resolve in three to five days or if the patient deteriorates. The patient was transferred to the regular floor for bowel rest and monitoring with serial abdominal x-ray while waiting few days for spontaneous resolution of the SBO. The patient was kept nothing by mouth. Serial abdominal x-ray in the supine and standing positions (Figures 3A, 3B) over a period of three days showed worsening of the bowel obstruction and were correlated with elevated levels of creatinine reaching a value of 3 mg/dL.

**Figure 4:**
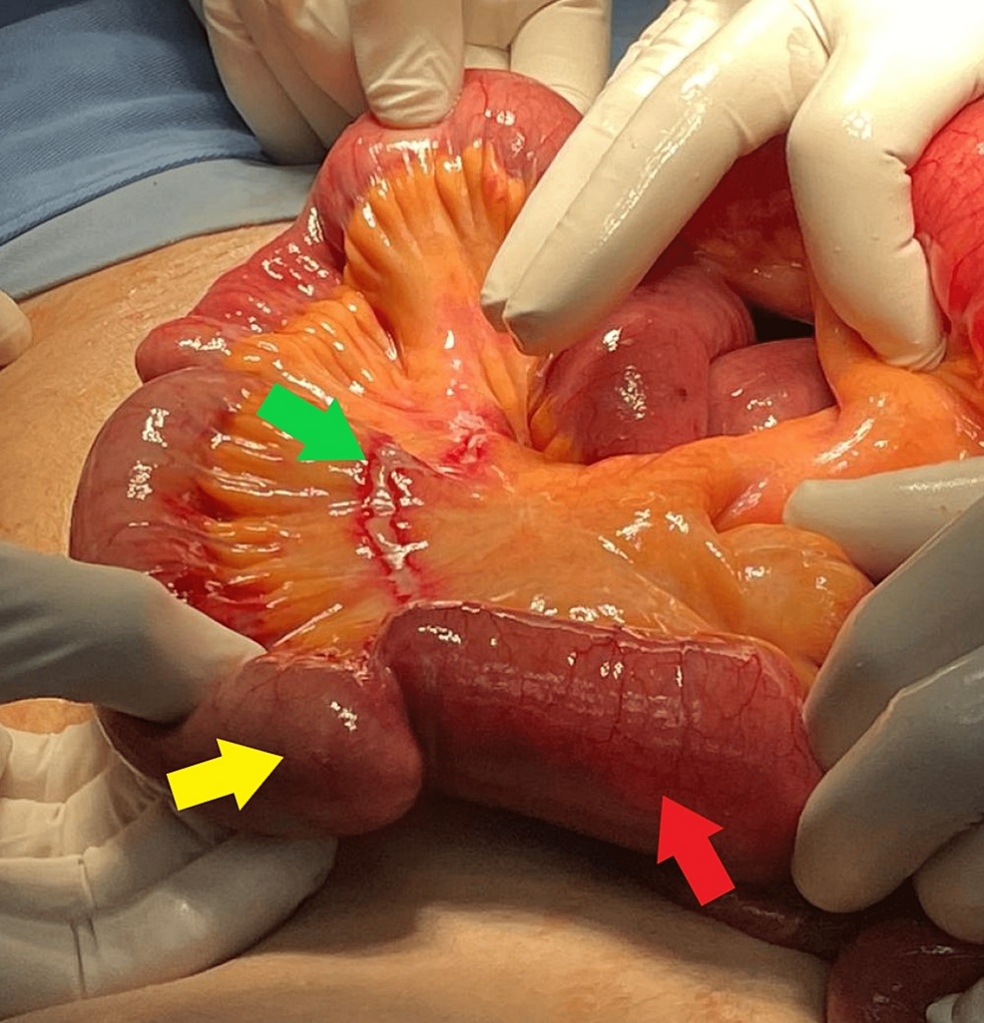
Picture from the laparotomy showing the Meckel’s diverticulum located in the terminal ileum, and a band of ischemia at the location where the adhesion caused the obstruction around the diverticulum (picture taken post-adhesiolysis). Yellow arrow: Meckel’s diverticulum; Green arrow: band of ischemia; Red arrow: terminal ileum

**Figure 5:**
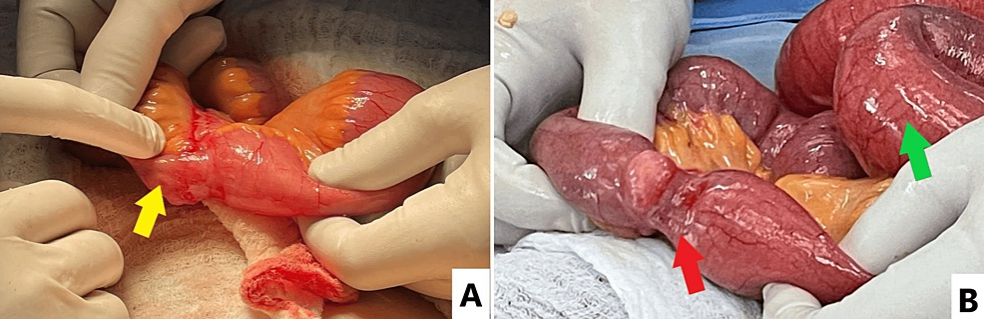
Pictures during the laparotomy showing the Meckel’s diverticulum in different angles. Yellow arrow: Meckel’s diverticulum; Red arrow: strangulation around insertion of diverticulum; Green arrow: distended terminal Ileum

On day 3 of hospital admission, the patient was transferred to the operating room for an exploratory laparotomy. During the operation, distended small bowels were identified with a transition zone in the distal ileum. An adhesion was identified along with a band of ischemia around a diverticulosis measuring around 1.5 cm presumed to be MD (Figures 4, 5A, 5B). A solid abnormality was palpated around the tip of the diverticular structure. Adhesiolysis was performed. A small segment measuring 5 cm containing the diverticular structure was resected and the small bowels were emptied. End-to-end anastomosis using Vicryl 3-0 and 4-0 interrupted sutures was performed, a lamellated drain was placed and closure done in three layers.

Pathology results of the specimen suggested Meckel's diverticulum (MD) with foci of inflammation and ectopic hypertrophic pancreatic tissue, there were no signs of malignancy. Post-operative course was non complicated. The patient improved clinically, inflammatory markers and creatinine started trending down at day 1 post-operation and the patient was able to drink clear fluids on day 3 and was discharged day 4 post-operation.

## Discussion

MD is the most common congenital malformation occurring in the gastrointestinal tract. It results from the incomplete obliteration and persistence of the vitelline (omphalomesenteric) duct during embryology. MD is a true diverticulum that contains all three layers of the intestinal wall and has its own blood supply from the superior mesenteric artery [3]. Although there are many anatomical variations, MD has been described in many textbooks by the “rule of two's”: it presents in 2% of the population, in a 2:1 male-to-female ratio, it is found approximately 2 feet from the ileocecal valve and measures around 2 inches. There are two forms of ectopic tissues found inside the diverticulum (ectopic pancreatic and gastric tissues) which can cause symptomatic presentation usually around the age of two [3,4].

According to a recent systematic review, the complications arising from MD in adults include obstruction, inflammation, and hemorrhage and their frequency in symptomatic patients were 35.6%, 29.4%, and 27.3%, respectively. The most common form of ectopic tissue was found to be gastric tissue followed by pancreatic tissue [3,4]. And based on a review of 1,476 cases of MD identified during surgery over a period of 50 years, the clinical and pathologic features that were most commonly associated with symptomatic presentation included male sex, age less than 50, diverticulum larger than 2 cm, and presence of ectopic tissue [5]. SBO is a common complication of MD in adults generally due to several mechanisms including intussusception, volvulus with or without acquired fibrous bands, torsion, abdominal wall hernia (Littre’s hernia), Meckel’s diverticulitis, and inversion of MD [2,5].

Mechanical SBO due to mesodiverticular band of adhesion arising from MD in adults has not been widely described in the literature. We, therefore, present a review of the literature for the several separate cases of SBO caused by adhesive bands formed on top of MD in adult patients aged 50 and above who had no previous abdominal surgery. The literature review was conducted by searching for relevant articles on PubMed Central and Google Scholar databases, the following results were obtained and displayed in Table 1 [6-12]. Only very few reported cases of SBO caused by MD in adults with virgin abdomens aged 50 and above were found with only one being an elderly aged 65 or above (a 92-year-old female). We, therefore, present a unique case of MD complication in this age group.

**Table 1:** A literature review of similar cases of adults with virgin abdomen presenting with small bowel obstruction because of adhesions on top of Meckel’s diverticulum CT: Computed Tomography

Authors	Age	Sex	Symptoms	Imaging	Laparotomy findings	Management
Ying et al. (2020) [6]	50	Male	Three days of lower abdominal pain, vomiting, and distention	Abdominal CT scan: dilated bowels with air-fluid levels and transition point	4 cm Meckel’s diverticulum with meso-diverticular adhesion	Segmentectomy and hand-sewn anastomosis
Ebrahimi et al. (2021) [7]	56	Male	Two days of abdominal pain with obstipation and vomiting	Abdominal CT scan: Small bowel obstruction	Meckel’s diverticulum adherent to mesentery through a band of adhesion	Bowel resection and anastomosis
Dutta et al. (2009) [8]	55	Male	Three days of severe colicky mid-lower abdominal pain with nausea	Abdominal CT scan: complete mid to distal small bowel obstruction	Meckel’s diverticulum forming a fibrous band and causing small bowel to twist on it-self	Resection of Meckel’s diverticulum segment
Takura et al. (2021) [9]	56	Female	Abdominal pain and vomiting	Abdominal CT scan: closed loop obstruction in terminal ileum	Meckel’s diverticulum with mesodiverticular band causing internal herniation of small intestines	Separation of adhesion band and resection of Meckel’s diverticulum at the root using a stapler
Sarkardeh & Sani (2020) [10]	92	Female	Three days of crampy periumbilical pain with abdominal distention and vomiting	Abdominal X-ray: Air fluid levels and dilated bowel loops	Perforated Meckel’s diverticulum	Meckel’s diverticulum segment resection followed by end-to-end anastomosis
Skarpas et al. (2020) [11]	63	Female	Umbilical pain, and distended abdomen associated with nausea and obstipation	Abdominal Imaging: Small bowel obstruction	8 cm Meckel’s diverticulum that was inflamed at the tip and formed a symphysis band with the mesentery	Separation of adhesion band and resection of diverticulum with stapler
Arslan et al. (2020) [12]	63	Male	One day of severe abdominal pain with nausea, vomiting, and distention	Abdominal X-ray: air-fluid levels and small bowels distention	Inflamed Meckel’s diverticulum with mesodiverticular band causing strangulation and ischemia	Separation of adhesion band and resection of small bowel segment

MD rarely causes complications in adults and is largely asymptomatic. When they do occur, complications include gastrointestinal bleeding and SBO [3]. In our case, a symptomatic MD was incidentally found during the exploratory laparotomy of an 80-year-old patient with a virgin abdomen presenting for SBO. Based on the history, it is presumed that this patient’s MD has been asymptomatic for years and went uncomplicated up until the patient’s presentation at 80 years of age when an adhesive band attached to this diverticulum caused the SBO.

While it is obvious that a symptomatic MD that is causing inflammation, bleeding, or intestinal obstruction should be surgically removed, there is still controversy on whether to resect an MD that is asymptomatic and incidentally discovered during abdominal exploration [13]. The decision to resect an asymptomatic MD depends on the patient’s characteristic and is a case-by-case approach [5,13]. Resection of MD can be performed either laparoscopically or through an open laparotomy. A recent comparative study on 681 children with MD showed that laparoscopic resection had a shorter post operative length-of-stay compared to open laparotomy but no significant difference in morbidity and operative times [14]. Additionally, laparoscopic converted to open had increased operative time but no effect on morbidity or length-of-stay [14]. Another retrospective comparative study on 148 cases of MD showed that even though the laparoscopic approach had equivalent outcomes to open laparotomy, 27.4% of laparoscopic cases required conversion to an open laparotomy [15]. In our case, an open laparotomy was performed after the failure of non-operative management. The laparotomy was uncomplicated, and the post-operative length of stay was four days.

Resection of MD can be performed either through simple diverticulectomy or through resection of the small bowel segment that contains the diverticulum (enterectomy) and followed by end-to-end anastomosis. Small bowel resection is preferred when there is a palpable abnormality in the diverticulum, or when the diverticulum is short and has a broad base (head-to-base diameter ratio less than 2). In these conditions, small bowel resection ensures complete resection of the diverticulum along with the ectopic tissues [13,16]. In our case, we went for small bowel resection since the diverticulum was short, broad-based, and had a solid abnormality when the tip was palpated.

## Conclusions

MD is rarely symptomatic in adults. SBO due to MD in the elderly population is an entity that has not been widely described in the literature. We present a very rare case of SBO in an 80-year-old patient with no previous abdominal surgeries (virgin abdomen). After the failure of conservative management, the cause of obstruction was determined on laparotomy to be an adhesive band formed on top of an MD. The obstruction was relieved and the small bowel segment that contained the diverticulum was resected, and hand-sewn anastomosis was made. This case report supports the idea that MD can still be a cause of gastrointestinal pathology in the elderly population and is not only limited to infantile and childhood ages. MD should be considered in the differential diagnosis of SBO in the elderly age group after ruling out the other more common etiologies.
